# Adverse childhood experiences, sleep quality/duration and later-life lower extremity function among older adults in China: evidence from CHARLS

**DOI:** 10.1186/s40359-025-02396-7

**Published:** 2025-01-27

**Authors:** Jiaqiang Xiao, Xiaosheng Dong, Meng Ding, Tao Kong

**Affiliations:** 1 College of Physical Education, Shandong Normal University, Jinan, 250358 China; 2 Centre for Health Management and Policy Research, School of Public Health, Cheeloo College of Medicine, Shandong University, Jinan, China; 3 Key Lab of Health Economics and Policy Research, National Health Commission of China, Shandong University, Jinan, China

**Keywords:** Adverse childhood experiences, Sleep quality, Sleep duration, Lower extremity function

## Abstract

**Objective:**

This study aimed to explore the relationship between adverse childhood experiences (ACEs), sleep, and lower extremity function in older adults using a nationally representative cohort.

**Methods:**

This study included 4,439 participants aged 60 years or older (mean age: 67.2 ± 5.7 years) from the China Health and Retirement Longitudinal Study (CHARLS) 2015 national survey and the 2014 Life History Survey. ACEs, sleep duration, and sleep quality were assessed through self-report, and lower extremity function was measured using the Short physical performance battery (SPPB). The relationships between ACEs, sleep, and lower extremity function were analyzed using multivariate linear regression model and restricted cubic splines.

**Results:**

After adjusting for covariates, older adults with four or more ACEs exhibited worse lower extremity function compared to those with no ACEs (β: -0.175). 6–8 h of sleep was associated with improved lower extremity function (β: 0.119), while good sleep quality was also associated with higher lower extremity function scores (β: 0.177). Age-related differences revealed that the association between four or more ACEs and reduced lower extremity function (β: -0.431) was significant only in individuals aged 70 years and older. In the 60–69 years age group, the sleep duration of 6–8 h was significantly related to better lower extremity function (β: 0.150), however, in those aged 70 years and older, more than 8 h of sleep was associated with poorer function (β: -0.378). Furthermore, good sleep quality was associated with better lower extremity function in individuals aged 70 years and older (β: 0.246).

**Conclusion:**

ACEs, particularly household mental illness and parental disability, are associated with poorer lower extremity function in older adults. Normal sleep duration and good sleep quality are linked to better lower extremity function and may mitigate the negative effects of ACEs. However, these associations vary by age.

## Introduction

Lower extremity function is a key determinant of quality of life and independent living in older adults [1, 2]. Research indicates that impaired lower extremity function significantly increases the risk of falls [3], and approximately one-third of community-dwelling older adults experience falls annually, which are a leading cause of both death and disability in this demographic [4, 5]. In China, falls are the primary direct cause of death among older adults [6], with estimated direct medical costs of RMB 5 billion and social costs ranging from RMB 60 billion to 80 billion [7].Moreover, age-related decline in lower extremity function is associated with various adverse health outcomes, including hospitalization [8] and disability [9]. Therefore, identifying risk factors associated with lower extremity function is essential for promoting healthy aging.

Adverse childhood experiences (ACEs) encompass a range of traumatic or stressful events occurring before the age of 18, including abuse, neglect, and family dysfunction [10]. The prevalence of ACEs among diverse populations in China varies from 50.5 to 75.0% [11–13]. Study have shown that ACEs, such as maltreatment, can negatively impact physical health and function later in life [14]. Specifically, ACEs are associated with cognitive decline [15] and reduced physical functioning in later life [16]. However, one study suggested that older adults with ACEs are more prone to lower extremity dysfunction [17]. Conversely, another study found that ACEs do not directly cause increased physical limitations in adulthood [18]. A significant gap persists in the literature, with a shortage of nationally representative, high-quality studies examining the relationship between ACEs and lower extremity function in older Chinese adults.

Studies have shown that physiological changes linked to aging cause widespread sleep disturbances in the elderly [19]. Poor sleep quality or abnormal sleep duration not only disrupts the hypothalamic-pituitary-adrenal axis, resulting in hormonal imbalances [20], but also leads to elevated levels of inflammation [21, 22]. Furthermore, substantial evidence shows that short sleep duration is linked to reduced mobility [23] and increased disability [24], whereas long sleep duration is associated with decreased walking speed [23]. Poor sleep quality is linked to diminished physical performance [23] and increased disability [25]. However, some studies suggest that both insufficient and excessive sleep are linked to decreased lower extremity function [26], whereas other research shows that the increased incidence of lower extremity dysfunction is not associated with sleep duration [27]. A longitudinal study has found that poor sleep quality is associated with a decline in lower extremity function in the elderly [28], whereas a cross-sectional study found that poor sleep quality is linked to poor lower extremity function in the elderly men, but the relationship is reversed in women [29]. This indicates gender heterogeneity in the relationship between sleep quality and lower extremity function, and age-related heterogeneity requires further exploration. Therefore, further investigation into the relationship between sleep and lower extremity function is warranted. Evidence indicates that both ACEs and sleep influence muscle function [30, 31], but it remains unclear whether sleep modulates the relationship between ACEs and lower extremity function in older adults.

Based on this, this study aims to investigate the relationship between ACEs and sleep patterns with lower extremity limb function in older adults, using a nationally representative sample from the China Health and Retirement Longitudinal Study (CHARLS). Additionally, physical performance changes in Asian older adults exhibit significant age-related heterogeneity, as the study will analyze age-related heterogeneity in this relationship.

## Methods

### Study participants

Data for this study were obtained from the 2015 survey and the 2014 Life History Survey of the China Health and Retirement Longitudinal Study (CHARLS). CHARLS is a national longitudinal study conducted from 2008 to 2020, involving residents aged 45 years or older across 28 Chinese provinces. The study utilized a multi-stage, stratified, probability-proportional-to-size sampling methodology. It includes data on demographic background, health status and function, social and economic status, and retirement information [32]. The 2014 Life History Survey provided detailed information on respondents’ family history, health history, education history, wealth history, and work history. CHARLS received ethical approval from the Ethical Committee of Peking University (IRB00001052-11015), and all participants voluntarily provided informed consent.

**Fig. 1:**
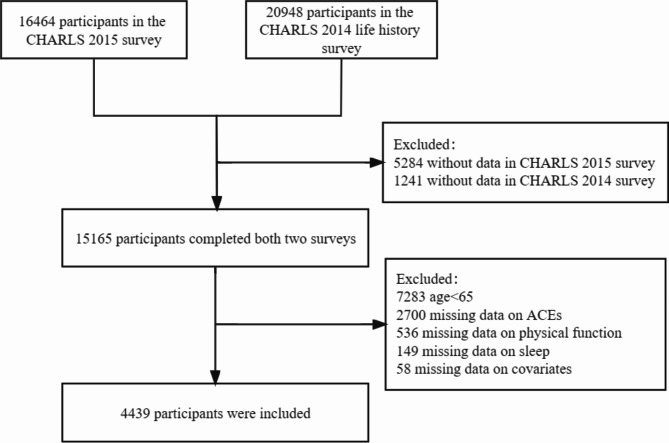
Flowchart of study participant selection

In this study, data from 16,406 respondents in the 2015 survey were matched with data from 20,948 participants in the 2014 Life history Survey. Participants were excluded if they met any of the following criteria: (1) age below 60 years, (2) missing data on adverse childhood experiences, sleep, and physical performance, or (3) missing information on covariates. Ultimately, 4,439 older participants (mean age: 67.2 ± 5.7 years) were included in the final analysis. Figure 1 illustrates the participant selection process.

### Lower extremity function

Lower extremity function was assessed using the Short Physical Performance Battery (SPPB). All participants completed the SPPB Chinese test following standardized instructions provided by a trained assessor. The SPPB consists of three components: walking speed test, repeated chair stand test, and balance test. Each component is scored from 0 to 4, leading to a total score that ranges from 0 to 12; higher scores indicate better lower extremity function [2].

1) Balance Test: The balance test includes semi-tandem, full-tandem, and side-by-side stands. Participants initially perform a semi-tandem stand, placing one heel beside the other foot’s toes. Those unable to maintain this position for 10 s proceed to side-by-side stands, with feet placed adjacent to each other. Those maintaining the semi-tandem stand for 10 s move to the full-tandem stand, with one heel placed directly in front of the other foot’s toes. Scoring reflects the duration of each stance: side-by-side for 10 s and semi-tandem for less than 10 s (1 point); semi-tandem for 10 s and full-tandem for 0–2 s (2 points); full-tandem for 3–9 s (3 points); full-tandem for 10 s or longer (4 points); or inability to perform the test (0 points) [33].

2) Walking Speed: Participants were instructed to walk along a 2.5-meter line twice at their usual walking pace, with the option to use canes or assistive devices as needed. The faster time from the two attempts was used for statistical analysis. Scoring criteria were as follows: ≤0.43 m/s (1 point), 0.44 to 0.60 m/s (2 points), 0.61 to 0.77 m/s (3 points), ≥ 0.78 m/s (4 points), and failure to complete the test (0 points) [33].

3) Repeated Chair Stand Test: Participants were instructed to sit in a standard chair (47 cm seat height) and cross their arms over their chest. They were required to stand up and sit down as quickly as possible, repeating the cycle five times consecutively without pausing or using their arms for support. The total time to complete the five repetitions was recorded. Scoring criteria were as follows: ≥16.7 s (1 point), 13.7 to 16.6 s (2 points), 11.2 to 13.6 s (3 points), ≤ 11.1 s (4 points), and failure to complete the test (0 points) [33].

### Adverse childhood experiences

Based on previous research [34, 35], we identified 12 ACEs indicators from the CHARLS dataset: physical abuse, emotional neglect, domestic violence, peer bullying, unsafe neighborhoods, parental death, parental disability, sibling death, household mental illness, substance abuse, parental separation or divorce, and incarcerated family members. Participants were categorized into three groups according to the cumulative number of ACEs: 0, 1–3, and 4 or more.

### Sleep duration and quality

Participants were asked, “In the past month, how many hours of actual sleep did you get each night on average?” (According to previous reports, the recommended sleep duration for middle-aged and elderly individuals in China is 6–8 h per day [36]). Based on their responses, sleep duration was categorized into three groups: short (< 6 h), recommended (6–8 h), and long (> 8 h). Additionally, participants’ sleep quality was assessed by asking about the number of days they experienced poor sleep, with responses classified as follows: none (< 1 day), some (1–2 days), occasional (3–4 days), and most (5–7 days). Sleep quality was further categorized as good (< 1 day), fair (1–2 or 3–4 days), and poor (5–7 days).

### Covariates

Based on previous studies [37, 38], the covariates in this study include: (1) demographics: age, gender, place of residence (urban and rural-urban or other), and employment status (employed or unemployed); (2) lifestyle: including current smoking status (yes or no) and current drinking status (yes or no); and (3) comorbidities: hypertension, diabetes mellitus, stroke, heart disease, chronic lung disease, and cancer (yes with one of these, no otherwise).

### Statistical analysis

Descriptive statistics were performed to summarize all socio-demographic characteristics, behavioral and lifestyle factors, and chronic disease data. Continuous variables were described using means and standard deviations, while categorical data were summarized with frequencies and percentages. Associations between individual ACEs, cumulative ACEs scores, and outcomes including sleep quality, sleep duration, and lower extremity function were examined using multivariate linear regression model. Three models were developed: Model 1, which excluded covariates; Model 2, adjusted for gender and age; and Model 3, adjusted for all covariates, including age, gender, residence, job status, smoking status, drinking status, and chronic diseases. Beta coefficients and 95% confidence intervals (95% CI) were calculated to report the strength of these associations. Additionally, stratification and interaction analyses were performed based on age groups (60–69 years vs. ≥70 years). Nonlinearity was explored using restricted cubic splines (RCS). Fully adjusted RCS linear analyses were conducted to assess the relationships between the number of ACEs and sleep outcomes, as well as their interactions with lower extremity function scores, stratified by age group. Statistical analyses were performed using STATA 17.0 (Stata Corp., College Station, TX, USA) and R version 4.3.3.

## Results

### Participant characteristics

Among the 4,439 participants included in this study, 2,292 (51.63%) were male and 2,147 (48.37%) were female, with a mean age of 67.2 ± 5.7 years. A total of 3,265 participants (73.6%) reported experiencing at least one ACE, and 245 participants (5.5%) reported experienced four or more ACEs. The majority of participants were from rural areas and reported no history of smoking or alcohol consumption. Participants who experienced four or more ACEs were more likely to be female and from rural areas than those who did not experience ACEs. (Table 1)

**Table 1:** Characteristics of participants by number of ACEs

Characteristics	Overall	No. of ACEs		
		0 (*n* = 1174)	1–3 (*n* = 3020)	≥ 4 (*n* = 245)
Age, mean (SD)	67.2(5.7)	66.7(5.4)	67.3(5.8)	67.6(6.5)
Gender				
Men	2292(51.63)	571(48.64)	1606(53.18)	115(46.94)
Women	2147(48.37)	603(51.36)	1414(46.82)	130(53.06)
Residence				
Urban and urban-rural	1101(24.80)	307(26.15)	741(24.54)	53(21.63)
Rural and other	3338(75.20)	867(73.85)	2279(75.46)	192(78.37)
Job status				
Employed	2676(60.28)	703(59.88)	1824(60.40)	149(60.82)
Unemployed	1763(39.72)	471(40.12)	1196(39.60)	96(39.18)
Smoking status				
Yes	1301(29.31)	331(28.19)	903(29.90)	67(27.35)
No	3138(70.69)	843(71.81)	2117(70.10)	178(72.65)
Drinking status				
Yes	1534(34.56)	380(32.37)	1068(35.36)	86(35.10)
No	2905(65.44)	794(67.63)	1,952(64.64)	159(64.90)
Chronic diseases				
Yes	2050(46.18)	533(45.40)	1401(46.39)	116(47.35)
No	2389(53.82)	641(54.60)	1619(53.61)	129(52.65)

### Relationship of adverse childhood experiences and sleep and their combinations to lower extremity functioning

**Fig. 2:**
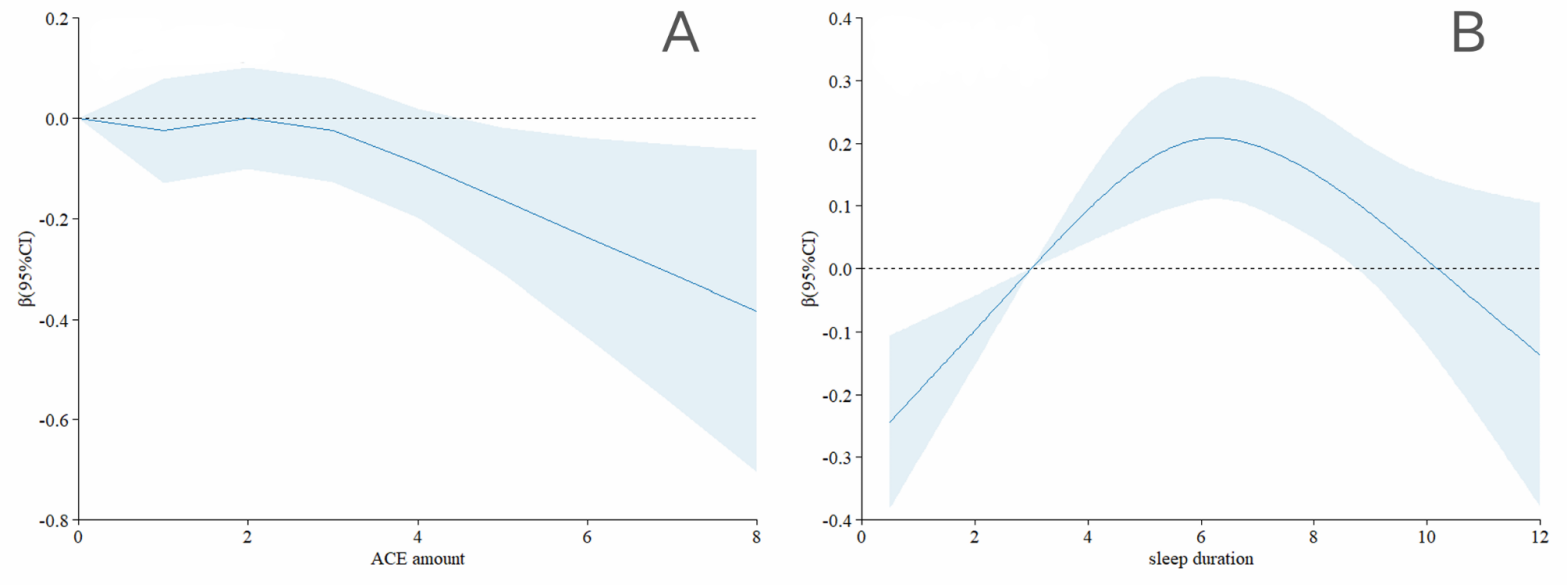
Association Between the Number of ACEs and sleep with lower extremity physical performance in restricted cubic spline model. The models were adjusted for age, gender, residence, job status, smoking status, drinking status, and chronic diseases

Table 2 presents the results of the multiple linear regression analyses examining the associations between ACEs and sleep and lower extremity function. In model 3, participants who experienced four or more ACEs had a significantly lower lower extremity function score by 0.175 (β: -0.175, 95% CI: -0.345 to -0.005) compared to those who did not experience ACEs. Findings for specific ACEs indicated that lower extremity function scores decreased by 0.111 and 0.122 for participants experiencing household mental illness and parental disability, respectively (household mental illness β: -0.111, 95% CI: -0.214 to -0.008; parental disability β: -0.122, 95% CI: -0.212 to -0.033). According to the restricted cubic spline model, lower extremity function scores decreased as the number of ACEs increased (Fig. 2A). Regarding sleep, lower extremity function scores were 0.119 points higher with 6–8 h of sleep compared to less than 6 h of sleep (β: 0.119, 95% CI: 0.039 to 0.198). Furthermore, participants with good sleep quality had higher lower extremity function scores by 0.177 points (β: 0.177, 95% CI: 0.082 to 0.272) compared to those with poor sleep quality. The restricted cubic spline model revealed an inverted U-shaped relationship between sleep duration and lower extremity function scores, indicating improvement with sleep durations up to 6 h and decline thereafter (Fig. 2B).

**Table 2:** Association between the ACEs and sleep with lower extremity physical performance

Variables	*N*	β (95% CI)		
		Model1	Model2	Model3
No. of ACEs				
0	1174	Ref	Ref	Ref
1–3	3020	0.005(-0.083 to 0.094)	0.013(-0.072 to 0.097)	0.013(-0.070 to 0.097)
≥ 4	245	-0.218(-0.398 to -0.037)*	-0.179(-0.351 to -0.006)*	-0.175(-0.345 to -0.005)*
ACE indicator				
physical abuse	1173	0.074(-0.014 to 0.162)	-0.006(-0.090 to 0.078)	-0.011(-0.093 to 0.072)
emotional neglect	1447	0.009(-0.073 to 0.092)	0.035(-0.044 to 0.114)	0.028(-0.050 to 0.106)
household substance abuse	319	-0.033(-0.183 to 0.117)	-0.039(-0.182 to 0.104)	-0.066(-0.207 to 0.075)
household mental illness	652	-0.135(-0.244 to -0.026)*	-0.119(-0.223 to -0.015)*	-0.111(-0.214 to -0.008)*
domestic violence	357	-0.112(-0.254 to 0.031)	-0.097(-0.232 to 0.039)	-0.083(-0.217 to 0.051)
incarcerated household member	22	0.298(-0.252 to 0.849)	0.235(-0.289 to 0.760)	0.231(-0.287 to 0.749)
parental separation or divorce	31	-0.184(-0.648 to 0.280)	-0.174(-0.616 to 0.269)	-0.167(-0.604 to 0.269)
unsafe neighborhood	340	-0.243(-0.388 to -0.098)*	-0.158(-0.296 to -0.019)*	-0.135(-0.272 to 0.002)
bullying	577	-0.021(-0.136 to 0.094)	-0.055(-0.165 to 0.054)	-0.060(-0.168 to 0.048)
parental death	1138	-0.068(-0.156 to 0.021)	-0.010(-0.095 to 0.074)	-0.005(-0.088 to 0.079)
sibling death	945	0.051(-0.044 to 0.145)	0.066(-0.024 to 0.156)	0.060(-0.029 to 0.149)
parental disability	924	-0.113(-0.208 to -0.018)*	-0.130(-0.221 to -0.039)*	-0.122(-0.212 to -0.033)*
Sleep duration				
< 6 h	1507	Ref	Ref	Ref
6–8 h	2478	0.235(0.151 to 0.318)*	0.139(0.058 to 0.219)*	0.119(0.039 to 0.198)*
> 8 h	454	-0.111(-0.248 to 0.026)	-0.106(-0.237 to 0.025)	-0.091(-0.221 to 0.039)
Sleep quality				
Bad	943	Ref	Ref	Ref
Fair	1193	0.118(0.006 to 0.229)*	0.100(-0.007 to 0.206)	0.083(-0.022 to 0.189)
Good	2303	0.291(0.192 to 0.390)*	0.207(0.111 to 0.303)*	0.177(0.082 to 0.272)*

* Significant at the 5% level

Figure 2 Association Between the Number of ACEs and sleep with lower extremity physical performance in restricted cubic spline model. The models were adjusted for age, gender, residence, job status, smoking status, drinking status, and chronic diseases.

**Fig. 3:**
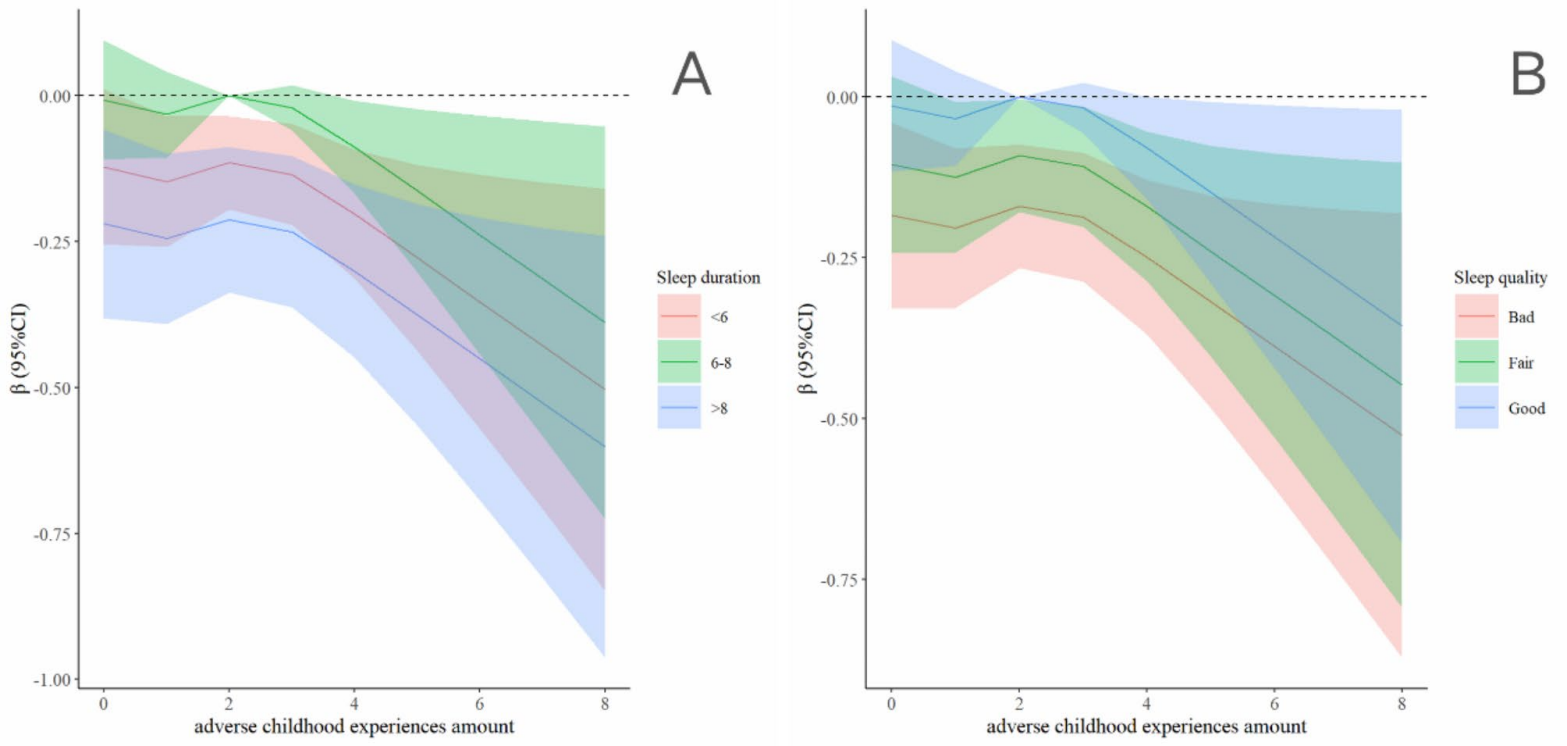
Association Between the combination of the ACEs and sleep with lower extremity physical performance in restricted cubic spline model. The models were adjusted for age, gender, residence, job status, smoking status, drinking status, and chronic diseases

Figure 3 depicts the restricted cubic spline illustrating the relationship between sleep duration, sleep quality, and ACEs with respect to lower extremity function. Among all participants, those sleeping between 6 and 8 h exhibited lower extremity function least impacted by ACEs, compared to those sleeping less than 6 or more than 8 h (Fig. 3A). Additionally, improvements in sleep quality were associated with a progressive reduction in the impact of ACEs on lower extremity function (Fig. 3B).

### Age differences in the relationship between adverse childhood experiences and sleep and their combinations with lower extremity function

Table 3 presents the results of a multiple linear regression analysis examining the relationship between the number of ACEs, sleep, and lower extremity function across different age groups. The analysis indicated that age significantly modulated the association between ACEs, sleep, and lower extremity function (P for interaction = 0.000). Specifically, having four or more ACEs was associated with decreased lower extremity function (β: -0.431, 95% CI: -0.811 to -0.050) exclusively in individuals aged 70 and older, in comparison to those without ACEs (Table 3). Additionally, a sleep duration of 6 to 8 h was associated with better lower extremity function in the 60–69 age group (β: 0.150, 95% CI: 0.066 to 0.234). In contrast, within the 70 + age group, sleeping more than 8 h was associated with poorer lower extremity function (β: -0.378, 95% CI: -0.645 to -0.111) (Table 3). Additionally, good sleep quality was associated with better lower extremity function in individuals over 70, while sleep quality had no significantly effect on lower extremity function in the 60–69 age group (Table 3).

**Table 3:** Association between the number of ACEs and sleep with lower extremity physical performance grouped by age

Variables	*N* (age 60–69/≥70)	Age group		*P* for interaction
		60–69	70 and above	
No. of ACEs				0.000
0	859/315	Ref	Ref	
1–3	2092/928	0.004(-0.083 to 0.090)	0.050(-0.144 to 0.244)	
≥ 4	169/76	-0.060(-0.240 to 0.119)	-0.431(-0.811 to -0.050)*	
Sleep duration				0.000
< 6 h	1021/486	Ref	Ref	
6–8 h	1813/665	0.150(0.066 to 0.234)*	0.052(-0.127 to 0.230)	
> 8 h	286/168	0.072(-0.071 to 0.214)	-0.378(-0.645 to -0.111)*	
Sleep quality				0.000
Bad	671/272	Ref	Ref	
Fair	838/355	0.156(0.045 to 0.266)*	-0.068(-0.308 to 0.173)	
Good	1611/692	0.246(0.146 to 0.345)*	0.025(-0.192 to 0.241)	

* Significant at the 5% level

**Fig. 4:**
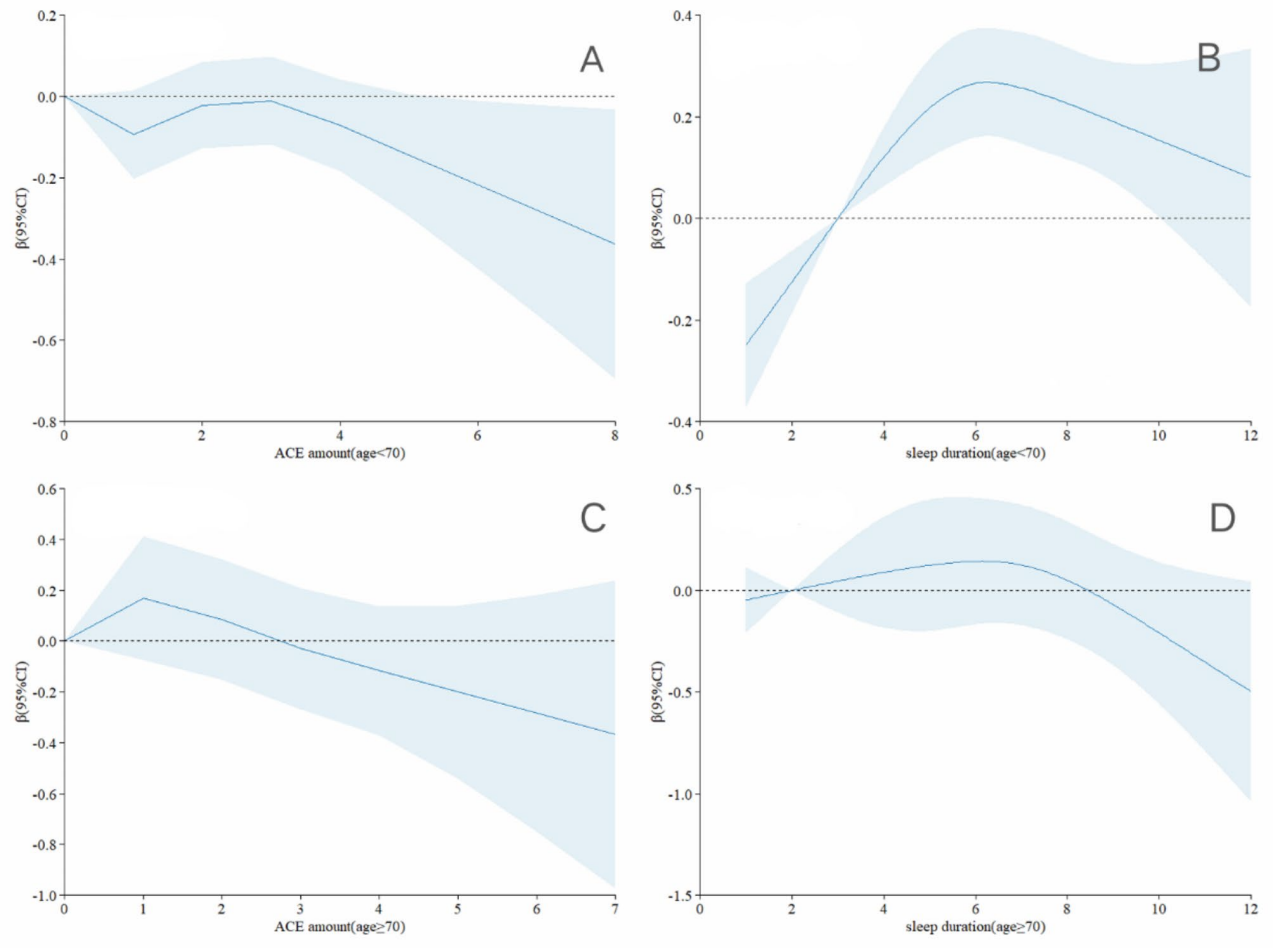
Association Between the Number of ACEs and sleep duration with lower extremity physical performance by age. The models were adjusted for gender, residence, job status, smoking status, drinking status, and chronic diseases

Figure 4 presents the results of the restricted cubic spline analysis regarding the relationship between the number of ACEs and both sleep duration and quality, as well as lower extremity function across different age groups. The analysis shown a positive correlation between having 0–3 ACEs and lower extremity function among participants aged 70 years or older, compared to those younger than 70 years (Fig. 4A C). Additionally, the relationship between sleep duration and lower extremity function exhibited an inverted U-shaped shape in both age groups. However, individuals younger than 70 years and those 70 years or older were more significantly affected by short sleep and prolonged sleep, respectively (Fig. 4B D).

**Fig. 5:**
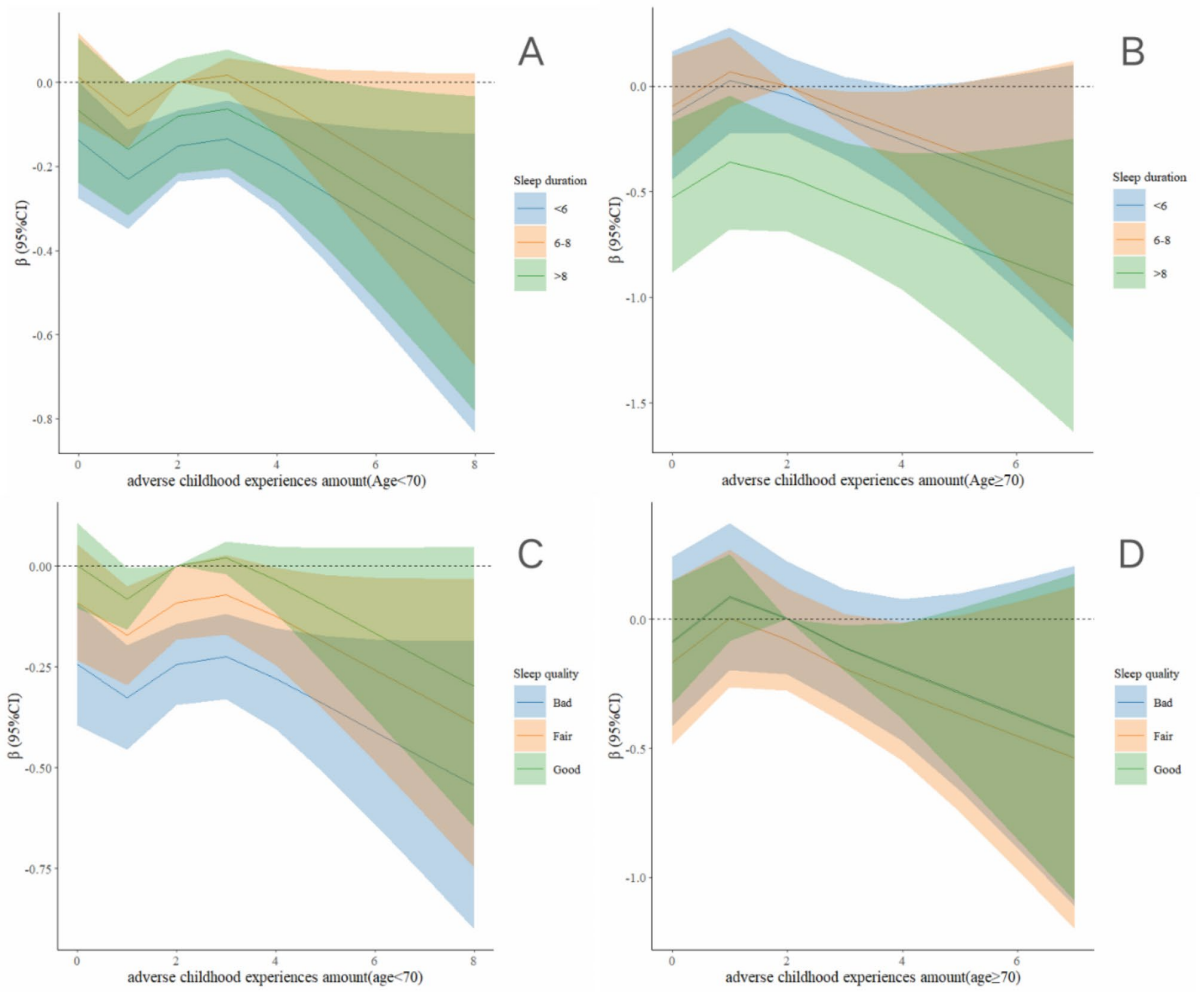
Association Between the combination of the ACEs and sleep with lower extremity physical performance in restricted cubic spline model grouped by age. The models were adjusted for gender, residence, job status, smoking status, drinking status, and chronic diseases

Figure 5 presents the results restricted cubic spline analysis, examining the relationship between ACEs, sleep duration, and lower extremity function across different age groups. In both age subgroups, participants who reported sleeping 6–8 h per night exhibited better lower extremity function relative to the number of ACEs, compared to those who slept less than 6 h or more than 8 h (Fig. 5A B). Additionally, among participants aged 60–69 years, those with high-quality sleep demonstrated the optimal lower extremity function relative to ACEs. Conversely, among participants aged 70 years or older, lower extremity function was similarly influenced by ACEs, regardless of sleep quality (Fig. 5C D).

## Discussion

The results of this study suggest that a higher number of ACEs (≥ 4) is associated with poorer lower extremity function in older adults (≥ 70 years), particularly in those with a history of household mental illness and parental disability. Moreover, the study found that a normal sleep duration (6–8 h per night) correlates with better lower extremity function, while both shorter and longer sleep durations adversely affect it. Additionally, older adults aged 60–69 years are more susceptible to the adverse effects of short sleep periods, while those aged 70 years and older are more vulnerable to prolonged sleep periods. Furthermore, good sleep quality is associated with better lower extremity function in older adults aged 60–69 years. The findings suggest that maintaining a normal sleep duration may mitigate the negative effects of ACEs on lower extremity function in older adults, while good sleep quality helps to counteract the decline in lower extremity function associated with ACEs in the 60–69 years age group.

The study found that a higher number of ACEs is associated with decreased lower extremity function in adults aged 70 years and older. This finding is consistent with previous research [17], which indicated that older Americans over the age of 50 who experienced ACEs are more likely to suffer from lower limb dysfunction. Studies suggest that ACEs may indirectly influence lower extremity function by fostering unhealthy behaviors, including smoking, overeating, and physical inactivity [39, 40], as well as by affecting brain structures such as the hippocampus [41–44]. Moreover, physical injuries sustained from childhood physical abuse could act as a risk factor for the decline in lower extremity function during later life [14]. Additionally, household mental illness and parental disability also contribute to the decline in lower extremity function. Studies have shown that parental mental health problems increase the risk of mental disorders in offspring, either due to genetic inheritance or disruption of the parent-child relationship [45], and these mental disorders are associated with a decline in physical function [46]. Parental disabilities not only pass on to their children [47]but also lead to inadequate responses to emergencies, posing a threat to the children’s safety and increasing the risk of accidents [48].

Our findings reveal an inverted U-shaped relationship between sleep duration and lower extremity function in older adults, indicating that 6–8 h of sleep per night is associated with optimal lower extremity function, consistent with previous research [26]. Existing research indicates that individuals that sleeping more than 9 h may experience a 15% reduction in weekly physical activity [49], negatively impacting lower extremity function [50]. Both short sleep duration and poor sleep quality impact muscle mass and strength via hormonal pathways related to muscle metabolism [51], are also associated with cognitive decline [52], further affecting lower extremity function [53, 54]. Notably, we find that younger older adults were more susceptible to short sleep durations, whereas older adults are more prone to the impacts of prolonged sleep. This age-related difference may be attributed to increased sleep disturbances with advancing age, which older adults often mitigate by extending their sleep duration [55, 56]. Additionally, better sleep quality was associated with better lower extremity function in individuals aged 60–69 years, but this association was not found in those over 70 years. This discrepancy may be attributed to older adults’ tendency to underreport sleep issues [30]. Consequently, it is recommended that older adults enhance sleep quality through modifications in physical activity to help prevent declines in lower extremity function [31].

We have found that normal sleep duration and good sleep quality are associated with a reduced negative impact of ACE on lower limb function, which we hypothesize may be related to muscle function. We have found for the first time that normal sleep duration and good sleep quality are associated with a reduced negative impact of ACE on lower limb function. ACEs may cause skeletal muscle damage and reduced muscle strength through increased inflammation and immune dysfunction [38]. Adequate sleep duration and good quality sleep are associated with improved muscle strength and mass by optimizing hormone-related muscle proteolysis, energy metabolism and maintaining physical activity levels [49–51]. However, the protective effect of sleep quality was not observed in adults over the age of 70, suggesting that age-related changes in sleep quality may affect its ability to protect individuals from early life adversity. Age-related changes in the neuroendocrine system that regulates sleep and stress responses may reduce the protective role of sleep against early life trauma [57]. Therefore, older adults who have experienced ACEs are recommended to aim for 6–8 h of quality sleep per night to mitigate the effects on lower extremity function.

This study has several strengths. First, we not only considered the total number of ACEs experienced by participants but also identified associations between specific types of ACEs and lower extremity function. Second, we investigated whether age-related factors influenced the associations between ACEs, sleep, and lower extremity function. This study also has several limitations. First, the data on ACEs were collected through participant recall, which may introduce bias. Second, certain ACEs, such as sexual assault, were not included in the study. Third, due to the cross-sectional nature of our study, it is not possible to determine the temporal relationship between the variables. Fourth, since the sample is only from China, caution is required when extrapolating the results to other cultural contexts or countries. Fifth, failing to stratify the analysis according to sleep characteristics has limited the depth and richness of the article.

## Conclusion

ACEs is associated with poorer lower extremity function in older adults, especially household mental illness and parental disability. Normal sleep duration and good sleep quality are associated with better lower extremity function in the elderly. There was a positive association between normal sleep duration and good sleep quality and the negative effects of ACEs on lower extremity function in older adults. However, these associations had different age heterogeneity. It is recommended that policymakers prioritize addressing the long-term effects of Adverse Childhood Experiences (ACEs) on lower extremity function in older adults by implementing strategies to improve sleep quality and duration, which could help alleviate the negative impact of ACEs on physical health.

## Data Availability

The data for this article comes from the China Health and Retirement Longitudinal Study. Available from https://charls.pku.edu.cn/en/.
